# A balloon‐assisted endoscopic submucosal dissection using long colonoscope and guidewire

**DOI:** 10.1002/deo2.259

**Published:** 2023-06-15

**Authors:** Yu Watahiki, Kazumasa Kawashima, Takuto Hikichi, Tadayuki Takagi, Michio Onizawa, Naohiko Gunji, Chiharu Watanabe, Jun Wada, Yuka Oka, Yuko Hashimoto, Hiromasa Ohira

**Affiliations:** ^1^ Department of Gastroenterology Fukushima Medical University School of Medicine Fukushima Japan; ^2^ Department of Endoscopy Fukushima Medical University Hospital Fukushima Japan; ^3^ Department of Diagnostic Pathology Fukushima Medical University School of Medicine Fukushima Japan

**Keywords:** balloon‐assisted endoscopy, colonoscopy, endoscopic submucosal dissection, guidewire, overtube

## Abstract

Balloon‐assisted endoscopy enables stable endoscopic maneuverability. Balloon‐assisted endoscopic submucosal dissection (BA‐ESD) is useful in the treatment of proximal colorectal tumors where scope maneuverability is poor. Herein, we reported a case in which BA‐ESD was successfully performed using a long colonoscope with a guidewire, although the lesion could not be reached using the balloon‐assisted endoscopy technique with a therapeutic colonoscopy.

A 50‐year‐old man underwent a colonoscopy that revealed a tumor in the ascending colon. BA‐ESD was performed using a conventional therapeutic endoscope due to excessive intestinal elongation and poor endoscopic maneuverability. However, the transverse colon loop could not be reduced, and the total colonoscopy failed despite using balloon‐assisted endoscopy. The scope was then changed from a conventional colonoscope to a long colonoscope, inserted into the terminal ileum, and the loop was reduced. After the guidewire was placed at the terminal ileum and the long colonoscope was removed, a therapeutic colonoscopy with an overtube was inserted into the ascending colon without reforming the colonic loop, allowing safe BA‐ESD.

## INTRODUCTION

Endoscopic submucosal dissection (ESD) for proximal colorectal tumors is at times difficult because of the formation of intestinal loops during long procedures, leading to paradoxical movement. The efficacy of balloon‐assisted ESD (BA‐ESD) as an approach for proximal colorectal tumors has been reported.^1^ However, some cases fail to reach the lesion even with balloon‐assisted endoscopy (BAE) because of the intestinal loops and excessive intestinal elongation.

## CASE REPORT

**FIGURE 1:**
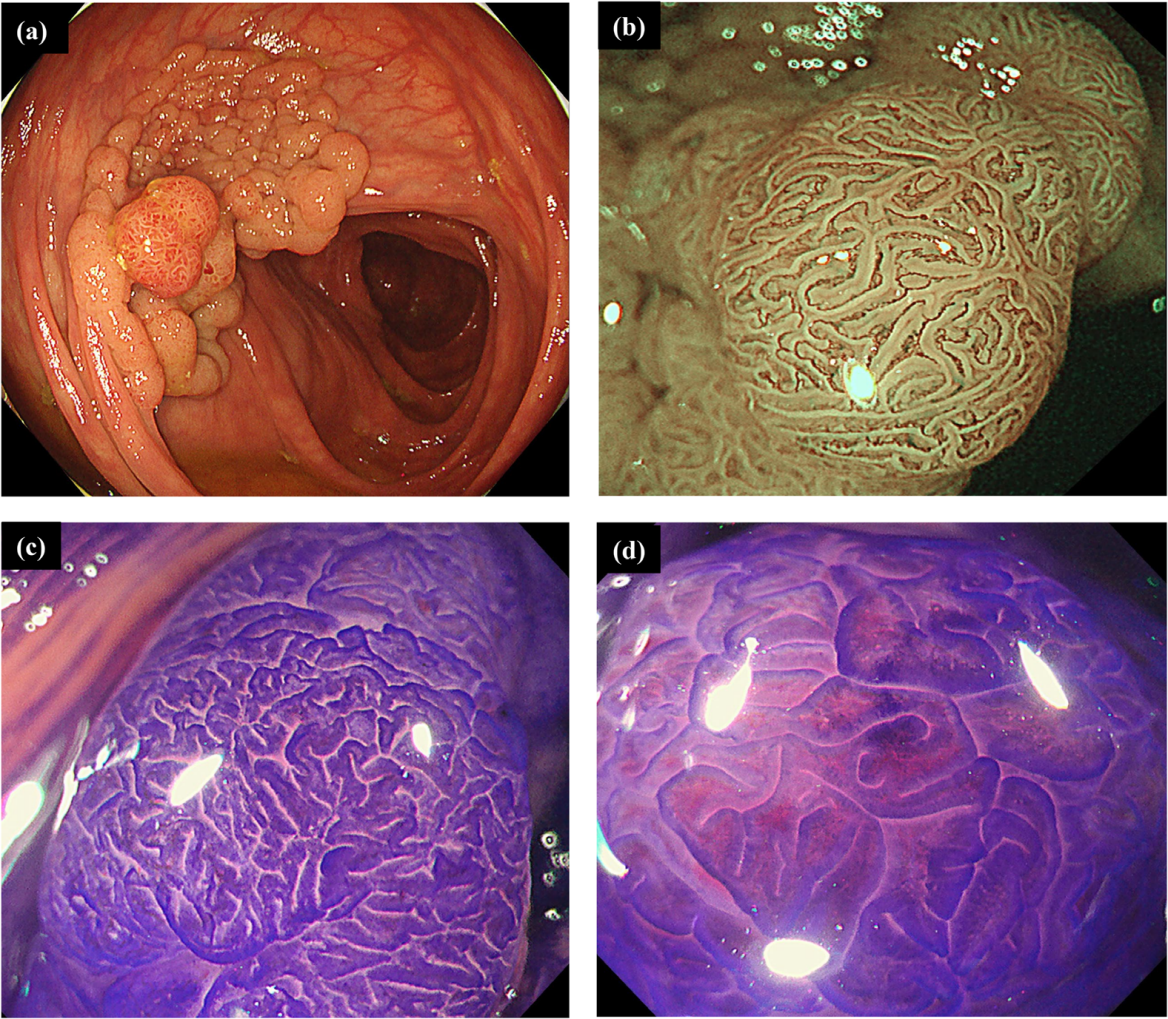
Colonoscopy findings (a) White‐light endoscopy revealed a 50 mm‐sized aggregation of a superficial elevated granular tumor with a semi‐pedunculated nodule in the ascending colon. (b) Magnified narrow‐band imaging at the nodule lesion revealed a Japan narrow‐band imaging classification of type 2A. (c) Kudo's pit pattern classification after crystal violet staining showed a type IIIL pit pattern in the superficial elevated granular lesion. (d) Kudo's pit pattern classification after crystal violet staining showed a type IV pit pattern in the nodular lesion.

**FIGURE 2:**
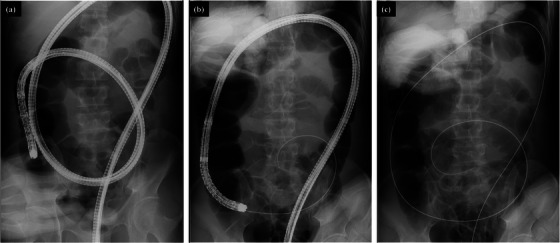
A long colonoscope and guidewire technique under X‐ray image. (a) Cecal intubation was successful using a long colonoscope. (b) After inserting the tip of the long colonoscope into the terminal ileum as deeply as possible, the loop of the transverse (c) Colon was reduced. A guidewire was inserted into the terminal ileum protectively and slowly through a colonoscopic biopsy channel while confirming the flow of the oral intestinal gas under X‐ray imaging. (d) To fix the tip of the guidewire retained in the ileum, the assistant inserted the guidewire while the endoscopist withdrew the colonoscope under X‐ray imaging.

**FIGURE 3:**
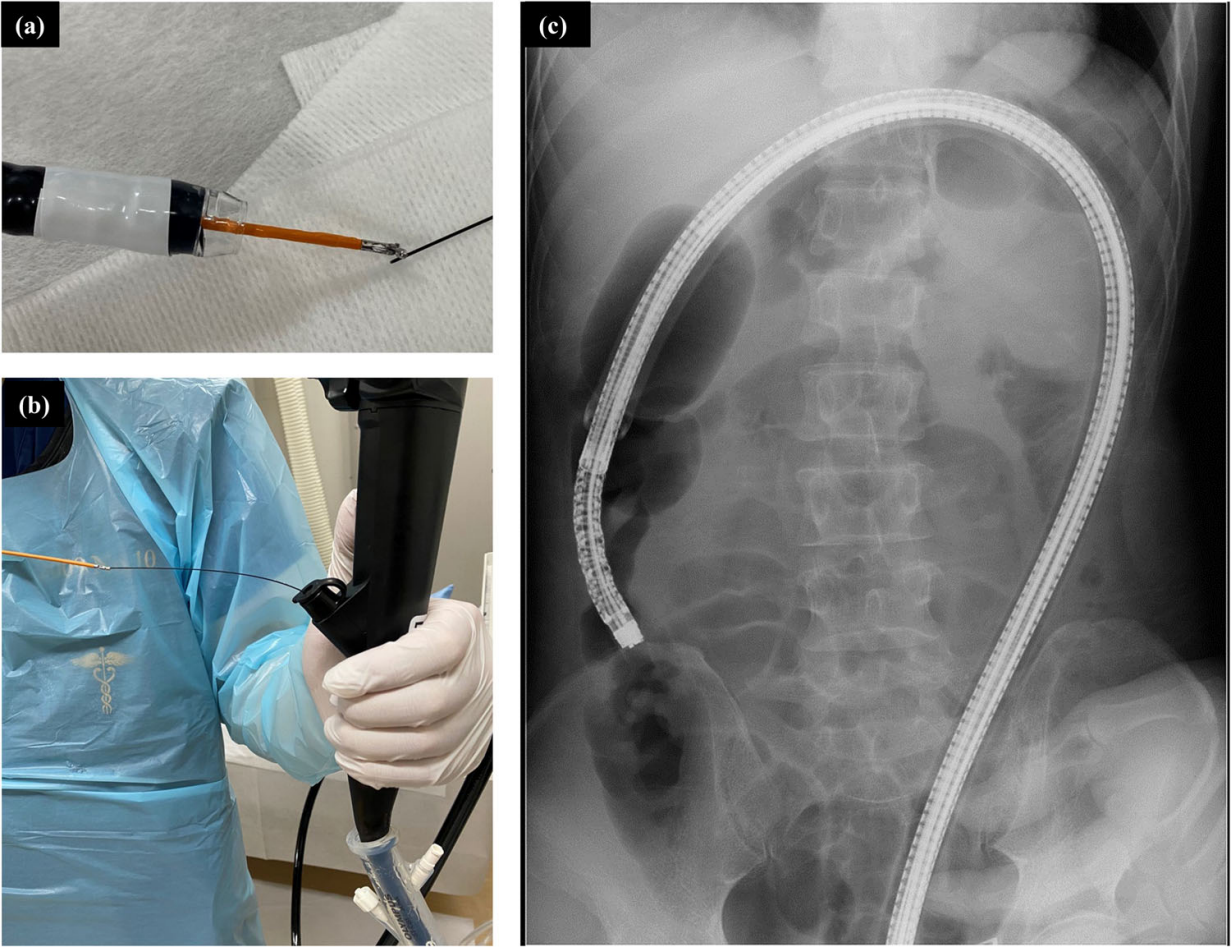
Balloon‐assisted endoscopy (BAE) using therapeutic colonoscope and overtube. (a) Biopsy forceps were inserted through the biopsy channel of the therapeutic colonoscope attached to an overtube, and grasped at the guidewire's anal end. (b) The guidewire was pulled out of the biopsy channel at the tip. (c) A therapeutic colonoscope with an overtube was then inserted without loop reformation around the guidewire, the entire overtube was inserted, and the balloon was fixed in the ascending colon.

**FIGURE 4:**
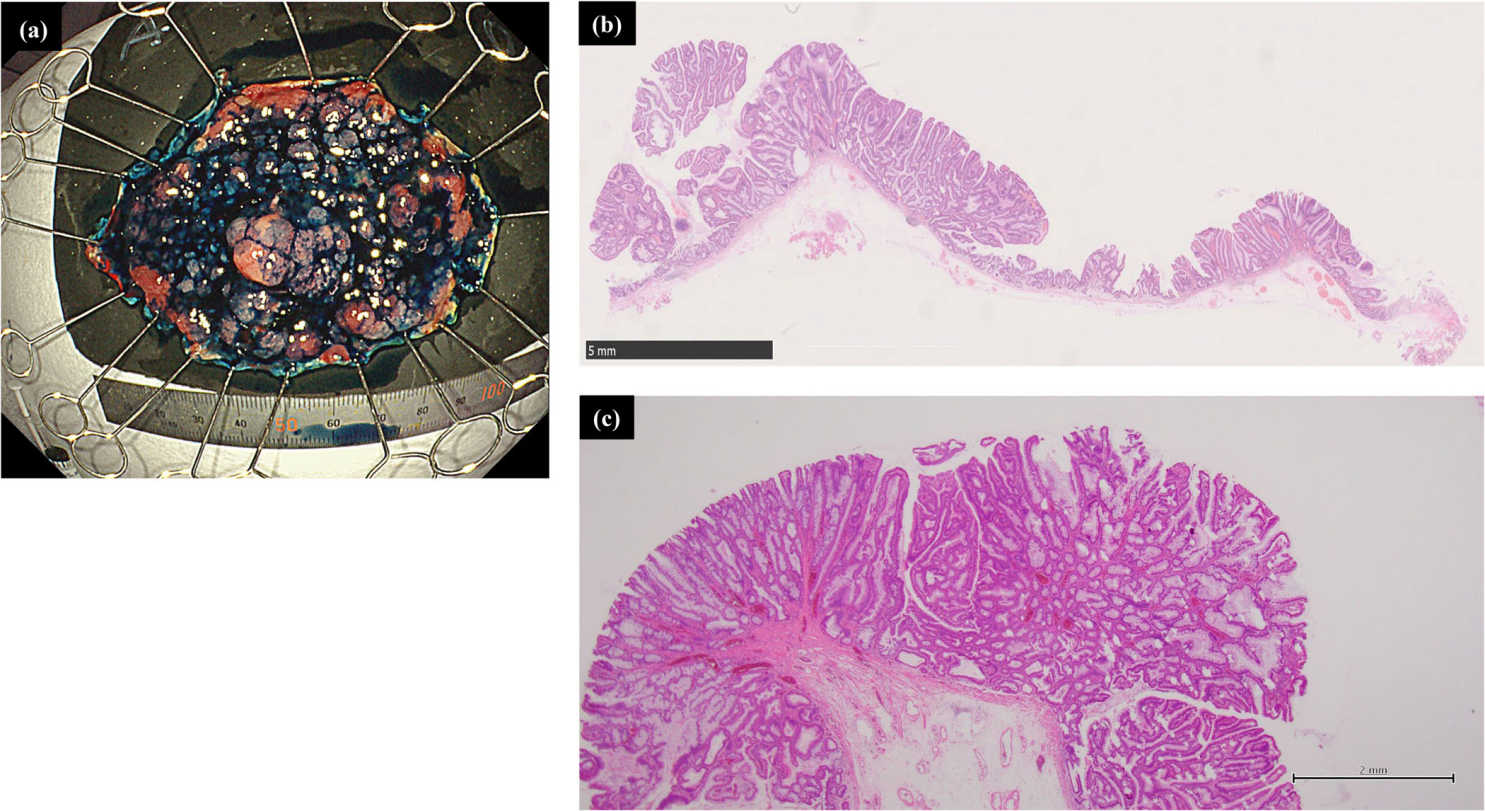
Specimen and histopathological findings. (a) Endoscopic submucosal dissection with good maneuverability, and en bloc resection were performed. (b) Histopathology showed an intramucosal tubular adenocarcinoma with negative horizontal and vertical margins. (c) There was no lymphovascular invasion in the high magnification.

A 50‐year‐old man presented to our institution with a positive fecal immunochemical test for colorectal cancer. Colonoscopy was performed using a magnified colonoscope (PCF‐H290ZI, Olympus Co., Tokyo, Japan) by an endoscopist with 14 years of experience, and cecal intubation was performed for 10 min. Colonoscopy revealed a 50 mm‐sized lesion in the ascending colon (Figure 1a). According to the Japan narrow‐band imaging classification, this lesion showed a type 2A pattern (Figure 1b), and Kudo's pit pattern classification after crystal violet staining showed types IIIL and IV pit patterns (Figure 1c,d). Based on these findings, we decided to perform ESD because of the possibility of early‐stage colorectal cancer. We also considered that BA‐ESD was necessary to safely remove the lesion because of the excessive intestinal elongation and poor endoscopic maneuverability during ESD. The ESD procedure was performed by a trainee with 8 years of experience in colonoscopy. Before performing ESD, a therapeutic colonoscope (PCF‐H290TI; Olympus Co.) with an overtube (ST‐SB1; Olympus Co.) was inserted under X‐ray imaging. However, the transverse colon loop could not be reduced, and the cecal intubation failed. The therapeutic colonoscopy and overtube were subsequently removed and replaced with a long colonoscope (PCF‐H290ZL; Olympus Co.), and the cecal intubation was successful (Figure 2a), and the transverse colon loop was reduced. A guidewire (VisiGlide2, 0.035Type; Olympus Co.) was inserted (Figure 2b), and the colonoscope was removed with the tip of the guidewire retained (Figure 2c). Next, biopsy forceps (Radial Jaw4P, Boston Scientific Co., Tokyo, Japan) were inserted, and the guidewire was grasped and pulled out through the biopsy channel (Figure 3a,b). The cecal intubation was successful without intestinal loop reformation, and an overtube was inserted into the ascending colon. (Figure 3c). The procedural time from the insertion of the long colonoscope to the initiation of BA‐ESD with a therapeutic colonoscope with an overtube took 25 min. The excellent maneuverability of the endoscope allowed safe ESD, and the lesion was resected en bloc without complications (Figure 4a).

The procedural time from the mucosal incision to the complete resection was 260 min. The resected specimen had a diameter of 77 × 48 mm, and histopathology revealed intramucosal tubular adenocarcinoma with negative horizontal and vertical margins and no lymphovascular invasion (Figure 4b,c). Based on these findings, curative endoscopic resection was performed. No delayed hemorrhage, perforation, or other complications were noted, and the patient was discharged on day 5 of hospitalization.

## DISCUSSION

Here, we reported a successful case of BA‐ESD for a colonoscopically difficult proximal colorectal tumor using a simple method with a long colonoscope and guidewire. This technique can be applied as a rescue procedure for patients in whom it is difficult to reach the lesion even with BAE.

The usefulness of the BAE method has been reported in cases with difficult cecal intubation. There are two types of BAE, including single BAE in which a balloon is attached to the overtube, and double BAE in which balloons are attached to the scope tip and overtube. Their cecal intubation rates were as high as 92.9%–98%^2^, ^3^ for single BAE and 96%–100%^4^, ^5^ for double BAE. However, in this case, cecal intubation was unsuccessful despite using the single BAE method. Difficult cecal intubation is reportedly associated with a history of abdominal surgery, colonic diverticulum, excessive colon size, female sex, low body mass index, and other factors.^6^ The patient was a medium‐built male with no history of abdominal surgery or colonic diverticula. In this case, several factors may have contributed to the cecal intubation failure. First, the thin therapeutic colonoscope made it difficult to transmit force and induced deflection of the intestinal tract, despite using the BAE method. Secondly, the intestinal elongation caused a transverse colonic loop, which made insertion more difficult. Preoperative colonoscopy was performed by an expert who has performed colonoscopy for 14 years, with more than 3000 colonoscopies and 100 colorectal ESDs.^7^, ^8^ In this case, it took the expert 10 min for cecal intubation, indicating this procedure was difficult.

In this study, a long colonoscope was more effective for difficult insertion cases with transverse colonic loops. The long colonoscope was inserted into the terminal ileum, which allowed the transverse colon loop to be reduced, and the guidewire was subsequently inserted into the ileum. The rigidity of the guidewire enabled deep insertion of the therapeutic colonoscope with an overtube without excessive intestinal elongation and loop reformation. A similar technique, endoscopic retrograde ileography, in which an overtube is inserted into the terminal ileum with a guidewire placed at the terminal ileum, has been reported to be useful in evaluating the terminal ileum in Crohn's disease.^9^ Shimizu et al. reported a case in which a forward‐viewing echoendoscope was inserted into the end of the afferent loop by preceding the guidewire to the reconstructed intestine after subtotal pancreaticoduodenectomy, allowing for transjejunal endoscopic ultrasound‐guided pancreatic drainage.^10^

An important finding from our case was that the method using a long colonoscope and guidewire may be an alternative technique for accessing the cecum in cases with difficult insertion, even when using BAE. Using this method, the therapeutic colonoscope and overtube can be inserted without intestinal loop reformation, and ESD can be safely performed under stable maneuverability.

In conclusion, we present the first case of BA‐ESD for a colonoscopically difficult proximal colorectal tumor using a long colonoscope and guidewire. A simple technique using a long colonoscope and guidewire are useful when cecal intubation cannot be achieved using BAE with a therapeutic colonoscope and an overtube.

## CONFLICT OF INTEREST STATEMENT

None.
